# Mechanisms of replication and repair in mitochondrial DNA deletion formation

**DOI:** 10.1093/nar/gkaa804

**Published:** 2020-10-06

**Authors:** Gabriele A Fontana, Hailey L Gahlon

**Affiliations:** Department of Health Sciences and Technology, ETH Zürich, Schmelzbergstrasse 9, 8092 Zürich, Switzerland; Department of Health Sciences and Technology, ETH Zürich, Schmelzbergstrasse 9, 8092 Zürich, Switzerland

## Abstract

Deletions in mitochondrial DNA (mtDNA) are associated with diverse human pathologies including cancer, aging and mitochondrial disorders. Large-scale deletions span kilobases in length and the loss of these associated genes contributes to crippled oxidative phosphorylation and overall decline in mitochondrial fitness. There is not a united view for how mtDNA deletions are generated and the molecular mechanisms underlying this process are poorly understood. This review discusses the role of replication and repair in mtDNA deletion formation as well as nucleic acid motifs such as repeats, secondary structures, and DNA damage associated with deletion formation in the mitochondrial genome. We propose that while erroneous replication and repair can separately contribute to deletion formation, crosstalk between these pathways is also involved in generating deletions.

## INTRODUCTION

**Figure 1.**
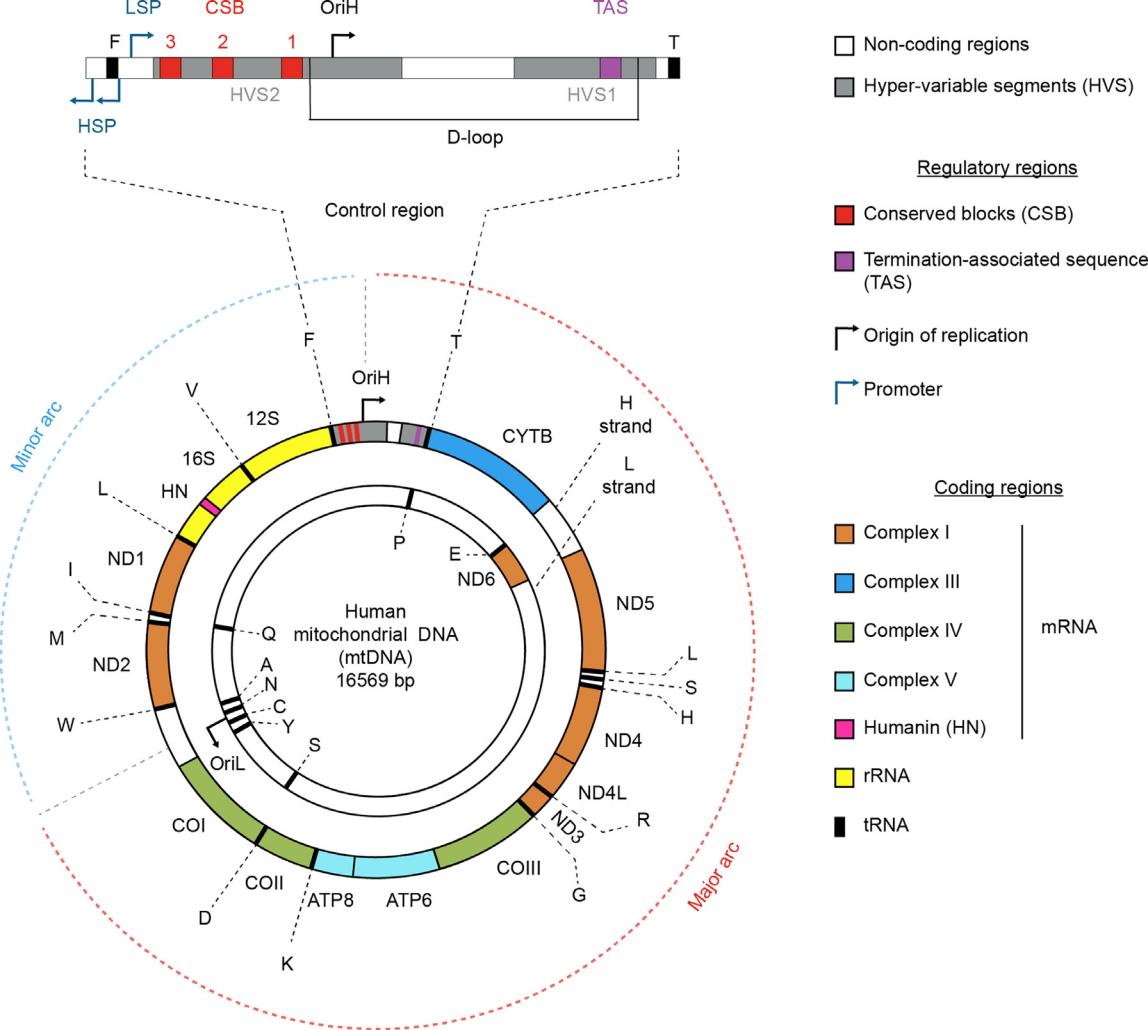
Schematic representation of human mtDNA, a double-stranded circular molecule of 16.5 kb. The H-strand and L-strand significantly differ in their base composition, with the H-strand containing a higher proportion of guanines. The mtDNA encodes for 13 proteins (colored boxes), the micropeptide Humanin (pink box), 2 rRNAs (yellow boxes) and 22 tRNAs (black boxes). With the exception of Humanin, all proteins encoded by the mtDNA genes are key components of complexes I, II and IV of the electron transport chain. Non-coding regions are depicted as white or grey boxes. A non-coding control region (expanded) contains crucial regulatory regions: three promoters (two H-strand promoters, HSPs and a L-strand promoter, LSP, shown as blue arrows), three conserved boxes (CSB 1–3, red boxes) and a termination-associated sequence (TAS, purple box). This non-coding region also contains the origin of replication of the H-strand (OriH, black arrow) and a triple-stranded displacement-loop (D-loop), a structure formed during premature termination of replication. The hyper-variable regions (HVS1–2, gray boxes) are highly polymorphic sequences and hotspots of germline and somatic mtDNA mutations. A second minor non-coding region is localized at approximately two-thirds of the genome and contains the origin of replication of the L-strand (OriL, black arrow). The mtDNA regions comprised between OriH and OriL shown as the major arc (red dotted line) and minor arc (blue dotted line) are depicted.

Mitochondria are descendants of α-proteobacteria and are responsible for energy production in most eukaryotic cells. Human mtDNA is a circular, 16 569 bp genome that contains 37 genes encoding for 13 proteins involved in oxidative phosphorylation, the Humanin micropeptide, 22 tRNAs and 2 rRNAs (1) (Figure 1). A 1211 bp non-coding region called the D-loop contains the heavy strand (H-strand) origin of replication (OriH), transcription factor binding sites and conserved sequence blocks. The light strand (L-strand) origin of replication (OriL) is located approximately two-thirds the circumference of mtDNA, starting from OriH. Depending on the type of cell, 100–1000s of mitochondria are present. Further, each mitochondrion harbors multiple copies (10–10,000) of mtDNA.

While mitochondria autonomously replicate their DNA, it remains elusive if mtDNA synthesis is coupled (2,3) or uncoupled (4) with the cell cycle. Mutations in this multi-copy genome are linked to a group of human diseases characterized by an overall decline in mitochondrial function and ATP production (5). The ratio of wild type to mutated levels of mtDNA in a cell, termed heteroplasmy, has a direct impact on healthy versus pathological status. Therefore, the increase in mtDNA mutational load contributes to mitochondrial disorders and is associated with mitochondrial dysfunction in cancer and aging. While point mutations are the most frequent mutational event in mtDNA, ∼250 mtDNA deletions have been reported (6–8). Moreover, large-scale deletions spanning kilobases in length contribute to genome instability and several pathogenic phenotypes (7,9).

## MITOCHONDRIAL DNA DELETIONS AND HUMAN DISEASE

Deletions in the mitochondrial genome are prevalent in several genetic disorders (5,10–12) including progressive external opthalmoplegia (PEO) (13), Pearson marrow-pancreas syndrome (PMPS) (14,15), and Kearns–Sayre syndrome (KSS) (13,16). A correlation of mtDNA deletions in cancer remains enigmatic (17). For example, in breast cancer, several studies show that deletion levels are higher in non-tumorigenic tissue compared to paired tumorigenic tissue (18–20), conversely, there is also data showing higher deletion levels in breast tumors (21). Concerning aging, levels of mtDNA deletions correlate with aging in several types of tissues, including brain (22–24), heart (23,25), skeletal muscle (26,27) and liver (28). Still, a causative link with cancer and aging to mtDNA deletions remains an open question. A high variability in the clinical manifestations of mitochondrial disorders has been described, and the onset of symptoms can range from infancy to adulthood affecting single or multiple tissues. It is generally accepted that these deletions are sporadic and less associated with maternal inheritance; yet, no unified view on how mtDNA deletions form was so far proposed. A study evaluating 226 families for a single large-scale deletion found the risk for offspring of an affected mother was ∼4% (29). Among the unique mtDNA deletions that have been described (7), the most frequently deleted genes are found on the major arc of mtDNA while, in contrast, the minor arc has the least deleted genes (Figure 1) (30). Furthermore, different deletions are associated with several human pathologies and their abundance directly affects the clinical presentation of a pathogenic phenotype (31).

Heteroplasmy and mosaicism, which is variation in the distribution of mtDNA polymorphic variants amongst tissue, present unique challenges to fully characterizing the role of mtDNA deletions in pathology. Heteroplasmy influences the onset of clinical symptoms and a pathogenic threshold is often ascribed to 60–90% of the mutant mtDNA (32,33). Overall, the onset and severity of disease is, in part, explained by mutational load and tissue distribution; however, an exact correlation is still lacking. Amongst the reported large-scale mtDNA deletions, the most prevalent in human disease is the common deletion (CD), a 4977 bp mtDNA deletion that is present in certain types of cancers and is associated with aging (22,34–36). This deletion was first described in 1989 in a patient with Kearns–Sayre syndrome/chronic external opthalmoplegia (16). In the case of mosaicism, there are examples reporting differences of CD levels based on anatomical location and tissue type (37). The generation of mtDNA deletions, including the CD, is poorly characterized on a molecular mechanistic level. Errors during mtDNA replication (16,38), dysfunctional mtDNA repair (reviewed in (9,39,40)), as well as intrinsic nucleic acid motifs contained within the mtDNA (41–44) were linked to the formation of deletions. While these elements make separate contributions in generating mtDNA deletions, potential crosstalk between the mtDNA replication and repair pathways could also be envisioned as a mechanism leading to deletions.

## MITOCHONDRIAL DNA REPLICATION AND DELETION FORMATION

**Figure 2.**
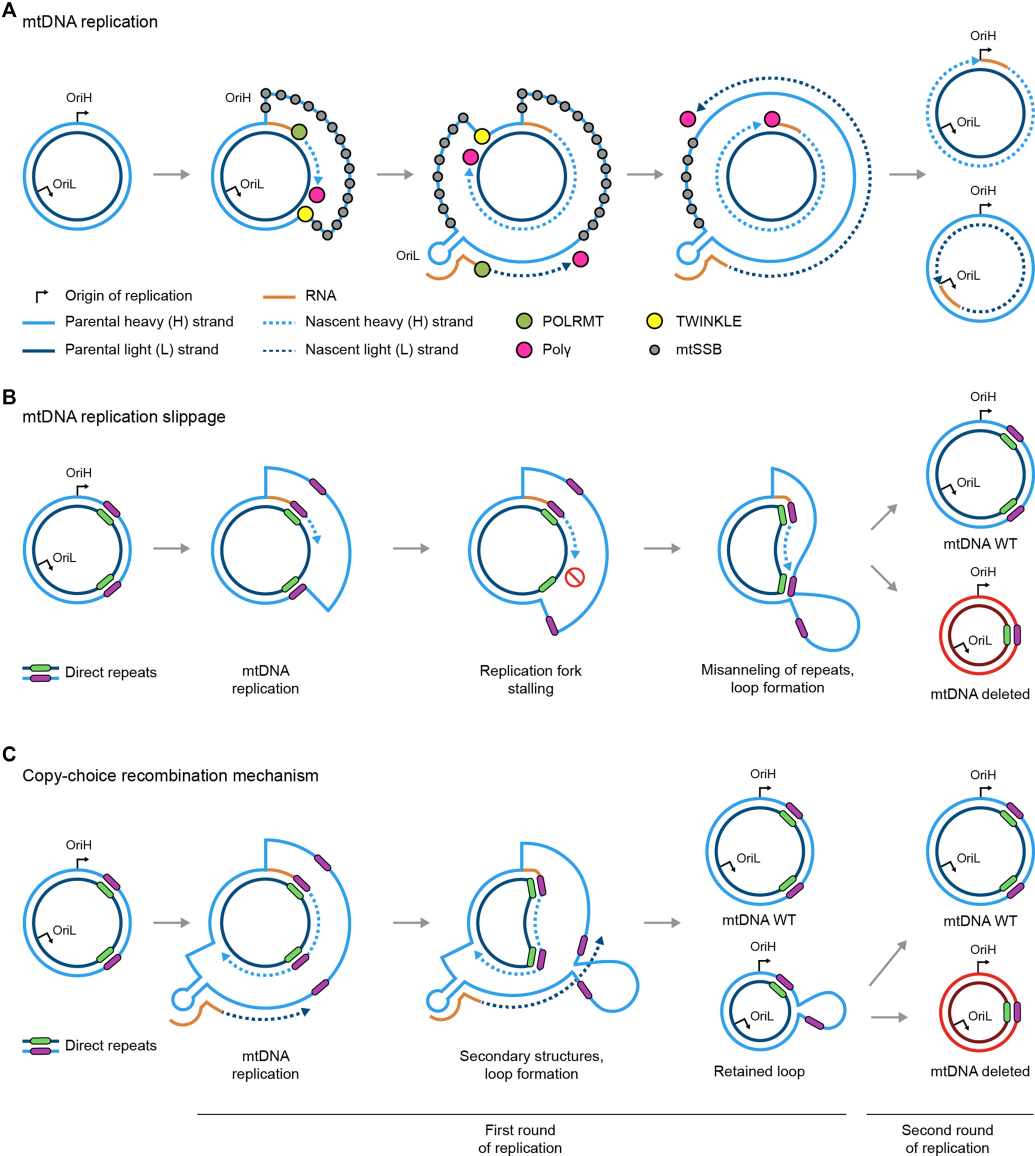
(**A**) Schematic of the strand displacement model (SDM) for mtDNA replication, which starts from two origins of replication, OriH and OriL, dedicated respectively to the replication of the H- and L-strand. In the SDM, mtDNA replication initiates from OriH. The mitochondrial RNA polymerase POLRMT synthesizes a short RNA sequences that primes the subsequent replication catalyzed by DNA Polγ. The Twinkle helicase progresses in front of DNA Polγ, unwinding DNA in an ATP-dependent manner. The exposed single-stranded H-strand is bound by mtSSB to prevent spurious replication events. Once the replisome reaches OriL, a single-stranded stem-loop structure is formed, blocking mtSSB binding and promoting the initiation of replication of the L strand. The stem-loop is recognized by POLRMT, which synthesizes a short RNA primer in the OriL region. DNA Polγ replaces POLRMT, and replication of the two strands proceeds unidirectionally and continuously to form two full double-stranded mtDNA daughter molecules. (**B**) Replication fork stalling is suggested to promote the mis-annealing of single-stranded mtDNA regions containing direct repeats. The loop generated during replication-slippage is extruded from the mtDNA molecule, resulting in deletions. (**C**) Deletion formation from copy-choice recombination involves replication starting from OriL. During replication, secondary structures and loops could be formed by the exposed single-stranded regions containing direct repeats. If those structures persist, the sequences comprised in this aberrant mtDNA conformation is deleted during the second round of replication.

Replication of mtDNA is distinct from the replication of the nuclear genome. The minimal mitochondrial replisome proteins reconstituted *in vitro* and found to perform mini-circle replication include DNA polymerase γ (Polγ), hexameric Twinkle helicase, and mitochondrial single-stranded binding protein (mtSSB) (45). Additional proteins that assist in mtDNA replication include mitochondrial RNA polymerase (POLRMT), transcription and packaging factor (TFAM), transcription elongation factor (TEFM), transcription factor B2 (TFB2M), exonuclease MGME1, DNA ligase III, and RNAse H1. The role of these replication factors in mtDNA synthesis is reviewed elsewhere (46). Of the three models proposed for mtDNA replication, the strand-displacement model (SDM) is the most accepted (46–48), although alternative mechanisms have been reported (reviewed in (49)). These models include coupled leading and lagging strand synthesis (50) and RNA incorporation throughout the lagging strand, termed RITOLS (51). In the SDM model, mtDNA replication is continuous from two distinct origins (Figure 2A). Replication starts at OriH and continues around approximately two-thirds of the genome (∼11 kb) until reaching OriL, whereby L-strand synthesis commences in the opposite direction resulting in unidirectional fork progression from each distinct origin. For L-strand initiation, POLRMT functions as a primase at OriL through recognition of a stem–loop structure (52,53). DNA Polγ replaces POLRMT, after ∼25 nucleotides, to commence mtDNA replication from OriL.

## MUTATIONS IN MITOCHONDRIAL REPLISOMAL PROTEINS ASSOCIATED WITH DELETIONS

Several mutations in nuclear-encoded proteins involved in mtDNA maintenance are associated with mtDNA deletions. For example, proteins related to mitochondrial dynamics (e.g. OPA1 and MFN2), nucleotide metabolism (e.g. TYMP) and nucleotide import (e.g. SLC25A4). These mutations and their clinical manifestations are reviewed elsewhere (54,55). Below, we discuss salient mutations in the mitochondrial replisomal proteins DNA Polγ, Twinkle and MGME1, encoded by the *POLG1*, *TWNK* and *MGME1* genes, respectively, that are associated with several human diseases and the accumulation of mtDNA deletions. DNA Polγ is a heterotrimeric protein comprised of the catalytic Pol γA subunit that interacts asymmetrically with the homodimeric Pol γB subunit (56). The Pol γA subunit displays both polymerase and proofreading activities, while the accessory Pol γB subunits function by increasing enzyme processivity during replication. Large-scale deletions were observed to significantly accumulate in mutator mice that harbor a proofreading-deficient mutation in the exonuclease domain of DNA Polγ (57–59). Several disease-associated mutations for the accessory Pol γB subunit are reported and correspond to multiple mtDNA deletions (60–62). For example, a C-terminal truncation (L475D variant) that renders the protein unable to efficiently bind to Pol γA was reported to cause replication stalling and accumulation of a mtDNA deletion found in PEO (63).

Dominant mutations for the Twinkle helicase are linked to adult-onset PEO (64). In fact, studies in transgenic mice that express Twinkle variants derived from PEO patient mutations show accumulation of multiple mtDNA deletions (65). The mutations expressed in these mice corresponded to two PEO patient mutations, the missense A359T mutation and the in-frame duplication 352–364 (A360T and 353–365 in mice). Structurally, the duplication mutation is the most severe mutation observed for Twinkle helicase. Further, this study evaluated the deletion breakpoints and found that the major deletion for the duplication mutant in muscle corresponded to a ∼13-kb mtDNA deletion. Work in human cells used CRISPR to engineer Twinkle R347Q and the homozygous Twinkle Y508C/Y508C mutant found to contribute to replication fork stalling (38). This observation corroborates earlier studies showing that fork stalling, by either Twinkle or Polγ mutations, is suggested to be an early step in mtDNA deletion formation (66–68). The dominant mutation R347Q is found in mitochondria disorder patients and resides in a linker region of the protein separating the primase-like and helicase domains (69). The recessive Y508C mutation resides within the helicase domain and is found in infantile onset spinocerebellar ataxia (70). By using a mtDNA fiber analysis assay, fork stalling was observed in the R347Q and Y508C/Y508C mutants in breakpoint regions relating to the mtDNA CD. While this suggests CD formation resulting from replication fork stalling, further work is needed to link a causal relationship and dissect the underlying molecular mechanism.

Cells deficient in MGME1, the mitochondrial exonuclease involved in processing mtDNA during replication, are associated with accumulation of a linear 11 kb fragment (71). The persistence of a linear mtDNA molecule is surprising, since linear molecules are often rapidly degraded (72,73), but this double-stranded mtDNA fragment is found to persist in MGME1 patient fibroblasts at a steady-state level ∼20%. It was proposed that this linear fragment is a byproduct of replication, possibly through aberrant ligation of the nascent H-strand at OriH and, thus, the creation of non-ligatable flaps near the origin of replication. Alternatively, it could imply that MGME1 participates in removing linear mtDNA, or potentially another mitochondrial nuclease may create single-stranded mtDNA as a substrate for MGME1. Similarly, *Mgme1* knockout mice accumulate multiple deletions as well as the long linear mtDNA fragments (74). These knockout mice were viable, but showed a replication stalling phenotype on mtDNA that was highly tissue specific.

## DELETION MECHANISMS ASSOCIATED WITH ERRONEOUS MITOCHONDRIAL DNA REPLICATION

The striking observation that most mtDNA deletions occur within the major arc, between OriH and OriL (Figure 1), could indicate that similar mechanisms are involved in forming these deletions. The first mechanism describing deletion formation was for the CD observed in KSS patients (16). In the CD, two 13-bp direct repeats flank the deletion breakpoint with only one of the repeats retained in the deletion construct, thereby suggesting a replication-slippage mechanism. The 13 bp 3′ repeat (13447–13459) and 5′ repeat (8470–8482) are hypothesized to mis-anneal during the displacement of the H-strand during replication. In the event of a break occurring on the parental strand downstream of the 3′ repeat, an intact 3′-hydroxyl would support continued mtDNA replication (Figure 2B). Subsequent degradation of the H-strand and ligation would result in the deletion. In support of this model, a recent study reporting a single-molecule fiber assay observed that single-strand breaks generated by mitoTALENS on the H-strand closest to the 5′ repeat lead to the formation of the CD. Conversely, nicks at both the 3′ repeat region and on the L-strand at the 5′ repeat region did not generate the mtDNA CD (38).

An alternative mechanism for deletion formation is the recently reported copy-choice recombination model, which originates from L-strand mtDNA replication (Figure 2c) (75). A similar mechanism has been demonstrated in *Escherichia coli* during lagging strand DNA synthesis when the template strand is single-stranded (76). To study the mechanism of copy-choice recombination, a circular template (∼4300 bp) containing a hairpin motif as a replication barrier was constructed. Following *in vitro* mtDNA replication reactions on the circular construct, PCR was performed to evaluate full-length vs. copy-choice recombination derived deletion constructs corresponding to PCR products of 1500 and 750 bp, respectively. The full-length 1500 bp product was generated in all conditions and, with increasing time, the 750 bp product was observed. In addition, experiments with increasing concentrations of mtSSB resulted in more full-length constructs and reduced the deleted mtDNA, suggesting a role for mtSSB in removing the hairpin replication barrier and subsequent stalling that would contribute to deletion formation. Overall, several mutations in mitochondrial replisomal proteins, e.g. Polγ, Twinkle and MGME1, contribute to deletion formation. What remains to be characterized is if the deletion formation process is more associated with replication errors originating from H-strand or L-strand synthesis. Besides replication-based mechanisms, repair of damaged mtDNA is also involved in deletion formation (40).

## MITOCHONDRIAL DNA REPAIR AND DELETION FORMATION

**Figure 3.**
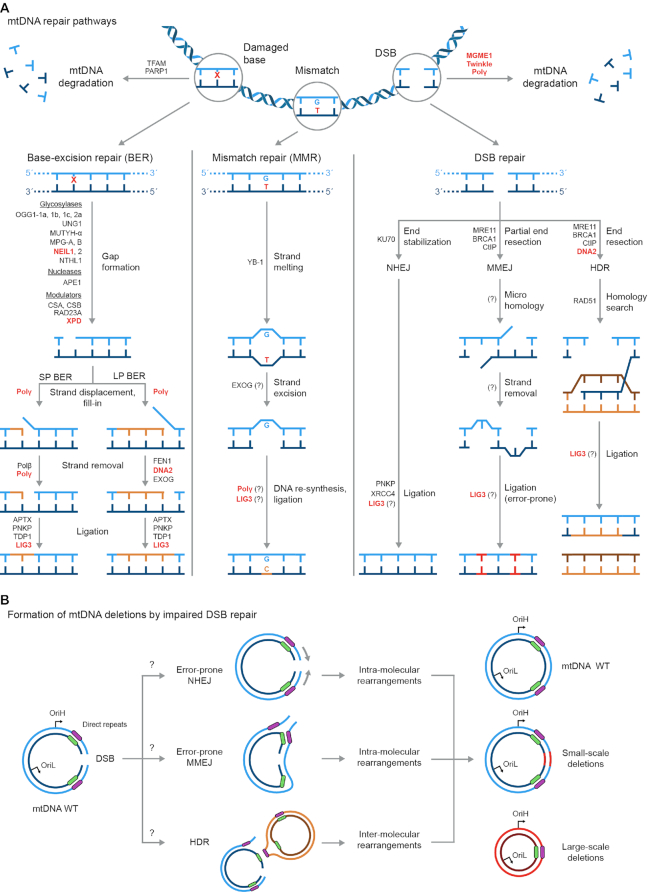
(**A**) Schematic representation of DNA repair pathways. While BER activity in mitochondria is highly characterized, MMR and DSB are still lacking substantive evidence. Repair proteins found in mitochondria are depicted in black, with proteins reported to play a role in deletion formation indicated in red. BER (left) repairs oxidized bases and abasic sites and comprises both short-patch (SP) and long-patch (LP) sub-pathways. MMR (middle) still lacks convincing evidence regarding its activity in mitochondria. YB-1, so far, is the only mitochondrial MMR protein identified. DSB (right) also lacks convincing evidence regarding efficient mitochondrial function. Proteins involved in mitochondrial non-homologous end-joining (NHEJ), microhomology-mediated repair (MMEJ) and homology-directed repair (HDR) were identified and appear as shared between the nucleus and the mitochondria; however, the mechanistic aspects of mitochondrial DSB repair, as well as the similarities or divergences with the nuclear pathways, are still largely unknown. In addition, mtDNA molecules harboring damaged bases and/or DSBs could also be degraded. (**B**) Hypothesized models concerning error-prone repair of mtDNA DSBs that lead to deletion formation, either by inter- or intra-molecular rearrangements. Potential mechanisms for the formation of small- and/or large-scale deletions could result from faulty re-joining of DNA ends during NHEJ (top), MMEJ (middle) occurring in presence of mis-annealed direct repeats, and unproductive HDR (bottom).

In comparison to the mechanisms regulating mtDNA replication, mtDNA repair pathways are less characterized. In addition, DNA repair mechanisms in the mitochondria are poorly resolved in comparison to nuclear DNA repair. Yet, it is becoming clearer that mitochondria display DNA repair activities, albeit with key differences compared to nuclear pathways (Figure 3A). While nuclear DNA is safeguarded by a repair-oriented maintenance, mtDNA molecules are either repaired or degraded. The selective depletion of damaged mtDNA lowers the overall population of mutated and/or deleted mtDNA molecules. For example, mtDNA molecules harboring abasic sites (77,78) or DNA double-stranded breaks (DSBs) (72,73) could be directly degraded rather than repaired. As mtDNA is under a strict copy-number control, selective depletion of damaged mtDNA molecules triggers replication of the undamaged mtDNA copies, reconstituting the mtDNA pool (reviewed in (79)). Besides selective depletion, damaged mtDNA can also be repaired.

In mitochondria, base excision repair (BER) is the most characterized mechanism to counteract the prominent generation of oxidized bases within mtDNA. Although mitochondria are considered to be devoid of nucleotide excision repair (NER), proteins involved in nuclear NER localize within the mitochondria (80–83) and are repurposed to function in BER. It is still debated whether mitochondria display efficient mismatch repair (MMR) and DNA double-strand break (DSB) repair activities and what relevance these pathways could have in mtDNA maintenance. However, proteins involved in these mechanisms have been found to localize in mitochondria (Figure 3A). Furthermore, dysfunctional repair of damaged bases and DSBs were suggested to be mechanistically connected to the formation of mtDNA deletions (9,39).

## BASE EXCISION REPAIR IS A BONA FIDE REPAIR PATHWAY IN THE MITOCHONDRIA

BER is involved in the repair of various types of DNA damage affecting the nuclear genome, including small non-bulky adducts, oxidized bases, as well as deaminated and hydrolyzed bases. In addition, single-strand breaks (SSBs) arising from abortive transcription or replication are also substrates for BER (84). The majority of BER proteins localize in both the nuclear and mitochondrial compartments; in addition, a subset of proteins involved in BER selectively localize within mitochondria, making mitochondrial BER highly efficient (Figure 3A, left). Further, alternative promoters and splicing patterns generate protein isoforms with selective sub-cellular localization, e.g. by encoding mitochondrial and nuclear localization signals, MLS and NLS, respectively. Glycosylases heavily rely on such regulatory mechanisms to constitute organelle-specific pools (85). For example, the human *OGG1* gene encodes the nuclear and mitochondrial OGG1-1a isoform and the mitochondrial-specific OGG1-1b, -1c and -2a protein variants (86–89), and the *UNG* gene produces the mitochondrial-specific UNG1 isoform and the UNG2 nuclear variant (90). Other BER proteins with MLS encoded in their isoforms are APE1 (91–93) and FEN1 (94). Main examples of proteins with exclusive mitochondrial localization are the ExoG nuclease, removing flaps generated in long-patch BER (93,95) and mitochondrial Polγ polymerase, fulfilling both replicative and repair activities within mitochondria (96,97).

Mitochondrial BER competes with the selective degradation of damaged mtDNA molecules (Figure 3A). Among the proteins that negatively regulate BER, TFAM binds the damaged mtDNA in close proximity to an abasic site. By steric exclusion of BER proteins, such as glycosylases and the APE1 nuclease, TFAM inhibits BER activation and promotes the degradation of the targeted mtDNA molecule (77,78). In stark contrast with its established roles in promoting nuclear DNA repair, the PARP1 protein inhibits BER within mitochondria. Under conditions of oxidative stress, PARylation of downstream mitochondrial BER proteins such as EXOG and Polγ leads to a decline in BER activity, potentially by decreasing the stability and/or the activity of enzymatic repair complexes (98). Mitochondrial BER is also modulated by the CSA, CSB (80,81), XPD (82) and RAD23A (83) proteins. These proteins play key roles in nuclear NER pathway, but function in BER once internalized within mitochondria.

The mechanistic links between dysfunctional mitochondrial BER and the formation of mtDNA deletions are not yet fully understood. Mice strains lacking mitochondrial BER were obtained by disrupting the predicted MLS of the OGG1 and MUTYH glycosylases. In such BER-deficient mice, no difference in the mutational load of the mitochondrial genome was detected, not even when the BER impairment was combined with increased oxidative stress (99). This observation suggested that a dysfunctional mitochondrial BER does not directly lead to mtDNA deletions. However, other studies found increased levels of mtDNA harboring deletion mutations in cells lacking BER proteins. For example, mice deleted for the gene encoding the glycosylase NEIL1 displayed a metabolic syndrome, and their liver cells showed increased mtDNA damage and signs of mtDNA rearrangements and/or deletions (100). Similarly, downregulation of the expression of the XPD helicase increases both the mtDNA oxidative damage and the levels of the CD in human cells (82). More evidence is required to establish a strong correlation between impaired mitochondrial BER and an increased incidence of mtDNA deletions. Still, these studies suggest that BER could safeguard mtDNA integrity and participate in decreasing the overall levels of mtDNA deletions. In fact, the repair of abasic sites and oxidized bases by BER may reduce the probability to develop mtDNA mutations, ensuring the maintenance of the genetic information encoding proteins of the electron transport chain. In this view, the proper functioning of the electron transport chain would ensure a low production of ROS, ultimately leading to less mutagenic events that could induce mtDNA rearrangements.

## EVIDENCE FOR MISMATCH REPAIR IN THE MITOCHONDRIA REMAINS ELUSIVE

While some evidence for mitochondrial MMR activity has been reported, it remains to be characterized whether this repair pathway functions within mitochondria. In the nucleus, the MMR pathway removes base mismatches, crosslinks and small insertions/deletions derived from DNA replication, recombination and repair events (Figure 3A, middle). Thus, MMR dramatically improves the fidelity of these DNA maintenance processes. MMR is defined as a strand-biased mechanism, as it relies on identifying the DNA containing an erroneously inserted base or insertion/deletion mispair (101). By analyzing the mtDNA mutations in samples from colorectal cancer, high levels of microsatellite instability were detected, indicating cancer-related MMR impairments (102). Additionally, an *in vitro* assay testing the repair of GT and GG mtDNA mismatches suggested the existence of MMR activity in lysates of rat liver mitochondria. However, mitochondria appear to be devoid of key proteins that carry out nuclear MMR, for example MSH2, MSH3, and MSH6 (103,104). So far, the multifunctional protein YB-1 is the only protein directly implicated in mitochondrial MMR. YB-1 recognizes and binds the mismatched DNA, melting the mtDNA strands and initiating repair. In addition, YB-1 interacts with the glycosylase NEIL2 and the nuclease APE1, suggesting that functional crosstalk between MMR and BER may exist for the maintenance of mtDNA (103).

Currently, there is no strong evidence supporting *bona fide* MMR within the mitochondria of mammalian cells. If such pathways do exist, there would be profound differences between nuclear and mitochondrial MMR. In fact, mitochondrial MMR would diverge from nuclear MMR as it lacks strand-bias. Therefore, mitochondrial MMR is unable to discriminate the strand containing the misincorporated nucleotide from the strand with the correct nucleotide (104). Based on this observation, it was proposed that mitochondrial MMR could be either a remnant of an ancestral pathway or a mechanism that merged with BER throughout evolution (105). It was also hypothesized that mitochondrial MMR may potentially help in resolving secondary structures in the mtDNA, with the aim to retain the DNA sequence comprised in hairpins and/or loops (105). However, so far, no studies have correlated MMR impairments to increased levels of mtDNA deletions.

## EMERGING EVIDENCE FOR MITOCHONDRIAL DOUBLE-STRAND BREAK REPAIR

DSBs are major threats to the stability of the nuclear genome, as they physically disrupt the continuity of chromosomes. Cells evolved two highly conserved pathways to repair DSBs occurring in the nuclear genome: homology-directed repair (HDR) and non-homologous end joining (NHEJ) (Figure 3A, right). HDR is based on the search for homologous templates to restore the integrity of broken DNA ends; conversely, NHEJ operates by re-ligating the two sides of blunt-ended DSBs (101). An alternative NHEJ pathway, named microhomology-mediated end joining (MMEJ), operates in absence of canonical NHEJ factors and relies on small regions of homology, usually from 5 to 25 nucleotides, to direct the re-ligation of broken DNA ends. As MMEJ leads to the formation of short flaps that are degraded prior to end joining, this pathway frequently leads to mutational events, especially small-scale deletions (106).

Evidence that mtDNA may be repaired through canonical DSB repair pathways were first reported in yeast (107–109). Still, whether efficient DSB repair is present in the mitochondria of mammalian cells remains an open question. The fact that mtDNA molecules harboring DSBs in mammalian cells are rapidly degraded by a mechanism involving the replication proteins Polγ, Twinkle, and MGME1 (72,73) indicates that mtDNA DSBs are handled differently than the repair-oriented mechanisms acting in the nucleus. However, experiments conducted *in vitro* with mitochondrial extracts or isolated mitochondria and *in vivo* with cell cultures suggest that mitochondria of mammalian cells may be capable of HDR (110–113), NHEJ (114,115) and MMEJ (116). Some DSB repair factors have been identified within mitochondria, for example MRE11 (117), RAD51 (118), DNA2 (119) and the NHEJ factors Ku80 (114) and XRCC4 (120) (Figure 3A, right). Overall, these findings provide indications, but no conclusive evidence, for the existence of efficient DSB repair activities in mitochondria. In addition, a limited number of DSB repair proteins have shown mitochondrial localization in mammalian cells. It is not yet clear how these isolated proteins could reconstitute DSB repair activities that are carried out in the nucleus by multi-protein complexes.

While the repair of broken mtDNA ends is still under investigation, links between unrepaired DSBs and the formation of large-scale mtDNA deletions are extensively reported (reviewed in (40,121)). The fast removal of broken mtDNA molecules could be considered a compensation for the lack of efficient DSB repair as well as a mechanism limiting the mutagenic potential derived from unproductive attempts of repairing mtDNA breaks. In fact, due to the heteroplasmic nature of mtDNA, the homology search needed to initiate HDR could promote erroneous recombination events, leading to deletions (Figure 3B). For example, the persistence of linearized mtDNA and the subsequent search for homologous sequences, among thousands of mtDNA molecules, lead to an increased incidence of detrimental inter-molecular mtDNA rearrangements (72). Similarly, the fact that mtDNA large-scale deletions are often flanked by short direct repeat sequences suggests that they could originate from the search of micro-homologies during MMEJ events (116). Alternatively, the attempt to repair linearized mtDNA molecules by error-prone NHEJ or MMEJ would frequently lead to intra-molecular recombination events, thus resulting in large-scale deletion formation (Figure 3B) (122,123). In conclusion, the existence of DSB repair activities within mitochondria remains unclear. Still, error-prone repair of DSBs evoked on the mtDNA and/or spontaneous recombination events could lead to mtDNA deletion formation, but more studies are warranted to dissect these mechanisms.

## NUCLEIC ACID MOTIFS IMPLICATED IN DELETION BREAK POINTS

**Figure 4.**
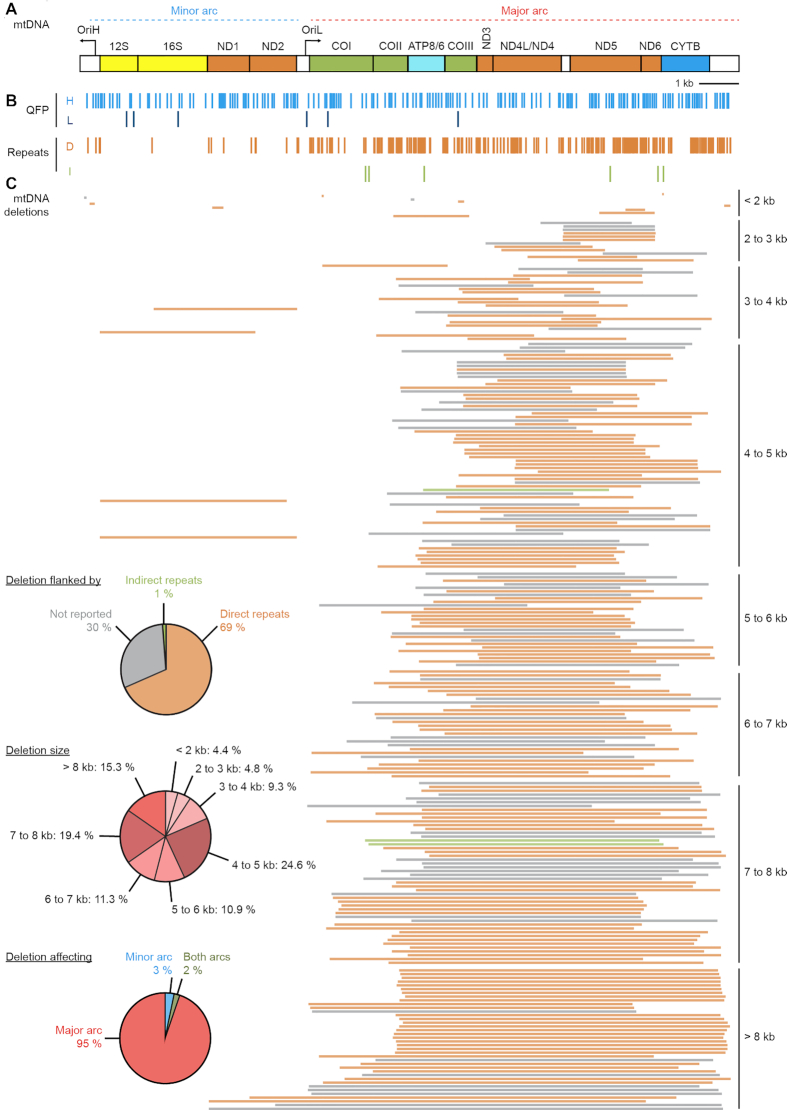
(**A**) Linear representation of human mtDNA. Genes are presented as colored boxes and non-coding regions as white boxes. tRNAs, regulatory elements and the distinction between genes localized on the H- or L-strand were omitted for clarity. (**B**) G4-quadruplex forming potential (QFP) sequences where computationally identified and mapped on the H- (light blue) and L- (dark blue) strands. Direct (orange) and indirect (green) repeats are indicated on the mtDNA as well as (**C**) 248 unique mtDNA deletions (accessed from Mitomap August 2020). Deletions are ordered by increasing size from top to bottom, ranging from 4 to 12 807 bp. Deletions flanked by direct repeats are indicated as orange lines, while deletion flanked by indirect repeats are indicated as green lines and deletions not yet reported to be flanked by direct or indirect repeats are instead depicted as gray lines. The top pie chart indicates the relative percentages of deletions flanked by direct, indirect, and flanking regions not yet reported to display repeats. The middle pie chart reports mtDNA deletion abundance based on size, while the bottom pie chart shows the percentages of deletions affecting the major and minor arcs or both.

Nucleic acid composition, including repeats, non-canonical structures and damage, play an underlying role in mechanisms of mtDNA deletion formation (Figure 4A–C). Based on break site sequence, mtDNA deletions can be categorized based on the presence or absence of repeats in their flanking regions. We analyzed the percentages of direct, indirect and instances of no repeats for the deletions reported in the online database Mitomap (7) (accessed August 2020). We calculated 69% of the reported deletions are flanked by direct repeats at the deletion break sites, with one repeat removed in the deletion construct. Instead, indirect repeats comprise 1% of deletions. The remaining 30% of cases involve deletions that do not have any repeat sequence reported. Direct repeats were the first nucleic acid sequence features identified to flank deletion break sites (41–44). For example, in the case of the CD, two direct 13 bp repeats are found at the deletion boundaries, with the 5′ repeat preferentially retained (43). This bias suggests that repeat sequences promoting intramolecular recombination are not the only contributing factor concerning deletion formation. Using the online Mitomap database (7), we mapped repeats against a linearized mtDNA molecule (Figure 4A and B). Indeed, there is an increased abundance of repeat sequences occupying the major arc in comparison to the minor arc as well as substantially more direct repeats in comparison to indirect repeats (Figure 4B and C). While repeats involve a sequence-based mis-annealing mechanism in deletion formation, they are not the only motifs implicated in forming deletions; it is also reported that secondary structures play an important role in deletion formation.

**Figure 5.**
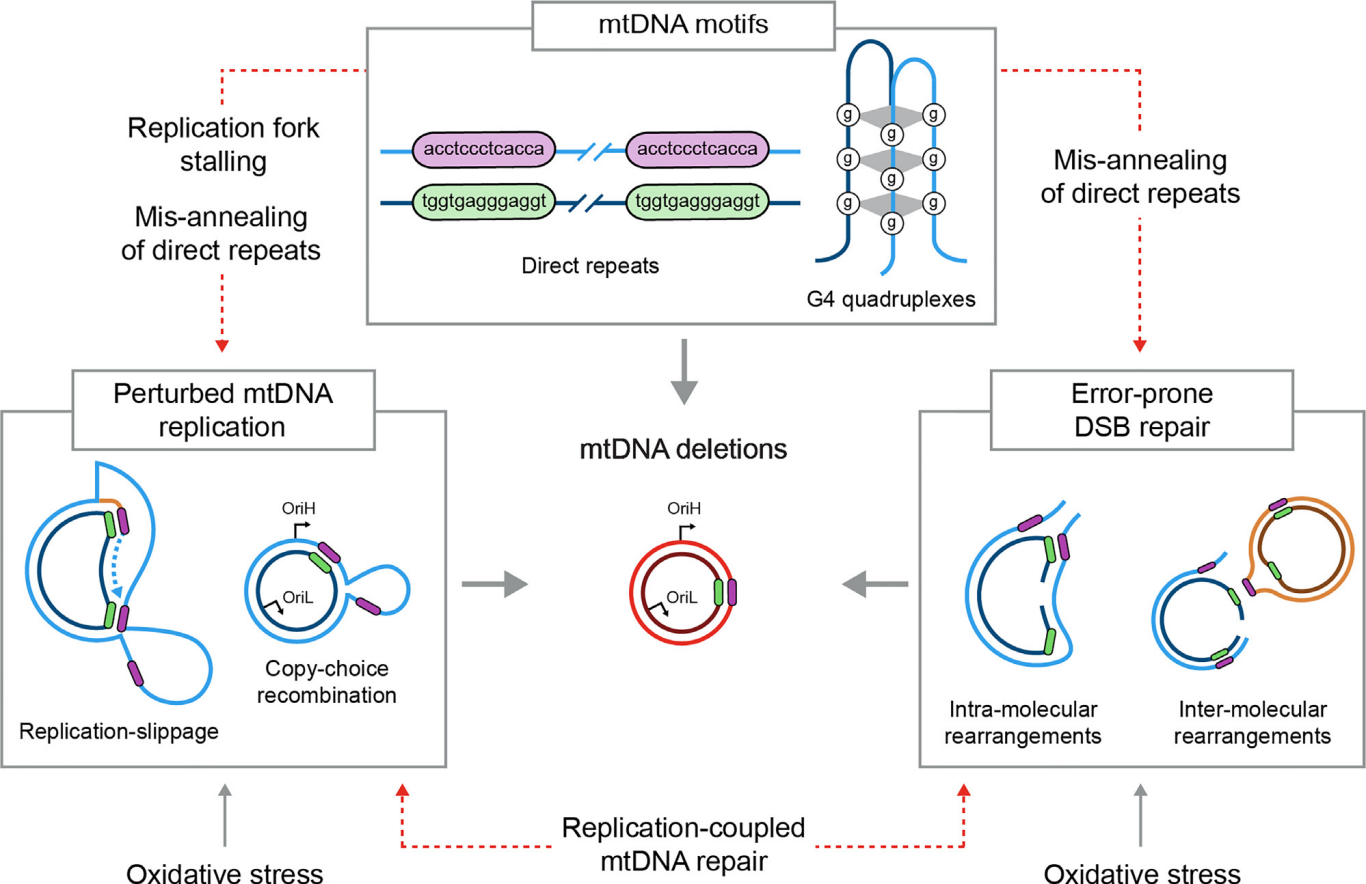
The main molecular determinants that participate in generating mtDNA deletions. mtDNA motifs, such as direct repeats and G4 quadruplexes, as well as mtDNA replication and repair pathways have been directly implicated in the formation of small- and large-scale deletions in the mitochondrial genome. In addition, crosstalk between replication and repair pathways (indicated as dotted red lines) have been described, particularly in formation of the CD where a replication-coupled mtDNA repair mechanism has been shown to cause accumulation of deleted mtDNA molecules. Mis-annealing of direct repeats has been linked to the perturbations in mtDNA repair and replication that ultimately lead to deletion formation. Similarly, replication fork stalling caused by G4 quadruplexes structures within the mtDNA could cause impairments in replication, leading to formation of loops and ultimately to deletions.

The mtDNA H-strand is rich in guanine bases in comparison to the L-strand, a sequence bias that could make the H-strand more prone to form G-quadruplexes (G4). Still, evidence is lacking to show if G4s are present in human cells. G4s are secondary structures characterized by planar stacking of four guanines called a G-tetrad. G4s form through non-covalent interactions of guanine nucleobases through Hoogsteen-based hydrogen bonding and are stabilized by a monovalent cation (K^+^ or Na^+^). Two or more G-tetrads can stack to form a thermodynamically stable quadruplex (Figure 5). G4s can adopt various orientations with varying degrees of stability (124–126). Further, G4 motifs are associated with genome instability, altered gene expression and have a demonstrated role in regulatory mechanisms (127).

Computational studies evaluating genomic regions with quadruplex forming potential (QFP) show that the mtDNA has a high tendency for G4 structures (Figure 4B). For example, G4 density is 2.4- to 3.6-fold higher in the mitochondrial genome compared to the nuclear genome (128). The higher proclivity for G4s in mtDNA and the reported role of G4s in deletion breakpoints suggests that mtDNA is inherently more unstable than the nuclear genome (127). We mapped predicted QFP regions (129) against linear mtDNA, which illustrates the higher amount of this secondary structure on the H-strand in comparison to the L-strand (Figure 4B). First work analyzing mtDNA G4s in *Saccharomyces cerevisiae* showed a 10-fold increase for G4s in mtDNA compared to nuclear DNA (130). Work mapping QFP in human mtDNA found more G4 motifs in humans than mouse, rat, and monkey (131). Further, 5′ and 3′ deletion breakpoint density was higher for QFP compared to tRNA regions. The analysis of deletion breakpoints in human disease showed association with neighboring QFP in PEO, KSS, PMPS, various cancers and aging (131).

The presence of G4s in mtDNA suggests a potential role in perturbation of DNA replication by fork stalling. To test this, *in vitro* unwinding assays with Twinkle helicase and G4 containing DNA substrates were performed. Twinkle was inefficient at unwinding well characterized G4 topologies, including a G4 substrate derived from mtDNA sequence for a deletion found in human renal cell carcinoma (131). While these data are compelling and provide mechanistic support for a role of G4 structures in deletion formation, what is currently lacking is convincing *in vivo* experiments to establish the presence and contribution of G4s in mtDNA deletion formation.

The role of mtDNA damage in deletion formation is currently unknown. While there is no direct evidence for damaged nucleobases (e.g. oxidized bases) as causal agents that promote deletions, it is interesting to speculate since oxidative stress is linked to deletion formation in the mitochondrial genome. The physiological source of DNA damage that promotes mtDNA breaks, and subsequent deletion formation, is poorly understood. However, both exogenous and endogenous pathways promoting oxidative stress lead to an increased frequency of mtDNA deletions. For example, the production of reactive oxygen species (ROS) as a by-product of oxidative phosphorylation has been linked to deletion formation due to the close proximity of the electron transport chain machinery to mtDNA (132). This observation could explain, for example, the increased incidence of deleted mtDNA molecules in aging tissues (133,134). Further, the decrease of the physiological functions of the electron transport and/or of the efficiency of ROS-scavenging enzymes has also been linked to deletion formation. For example, partial loss of the activity of the manganese superoxide dismutase (SOD2) in mice (135) and disruption of the adenine nucleotide translocator isoform 1 (ANT1) in mice and human lead to the accumulation of mtDNA deletions (136,137). Regarding exogenous sources of ROS, exposure to UVA radiation correlates to mtDNA deletions, likely through the oxidative damage of bases. For example, human skin fibroblasts treated with sub-lethal doses of UVA showed increasing levels of the CD. Further, the levels of the CD were reduced in the presence of singlet oxygen quenchers, demonstrating the importance of reactive oxygen species in the formation of the mtDNA CD by means of UV exposure (138). Still, the role of mitochondrial ROS and mtDNA deletion formation require further investigation to understand underlying molecular mechanisms.

## POTENTIAL ROLE FOR REPLICATION-REPAIR CROSSTALK IN MITOCHONDRIAL DELETION FORMATION

Molecular mechanisms for how deletions form in the mitochondrial genome remain an open question. To date, 248 unique deletions were reported in the Mitomap database (accessed in August 2020) (7), ranging from 4 to 12807 bp that prevalently localize in the major arc of the mtDNA (Figure 4C). Further, the majority of mtDNA deletions (∼60%) are greater than 5 kb in size (Figure 4C). There is not a united view for how these mtDNA deletions are generated; however, replication errors and aberrant repair contribute to mtDNA deletion formation. While these two mechanisms can function separately (9), it is feasible that crosstalk between these two pathways cooperate to generate deletions. The three main mechanisms suggested for mtDNA deletion formation include (i) replication-slippage, (ii) copy-choice recombination and (iii) aberrant DSB repair (Figure 5). The replication-slippage model, involves deletion formation based on the presence of single-stranded DNA during replication progression across the major arc (Figure 2B). The second model is the recently reported copy-choice recombination pathway that relies on replication initiation from OriL (Figure 2C). The third model involves DSB formation followed by aberrant repair (Figure 3B). Regarding the replication-slippage model, deletions with preferential retention of the repeat closest to OriH would occur, as in the case of the formation of the CD. Further, deletions that involve direct and indirect repeats would likely result from replication-slippage mechanisms that rely on directionality. Conversely, deletions that do not involve repeats are likely ascribed to aberrant repair pathways that can occur independent of this directionality.

An emerging picture shows that replication and repair are intimate pathways involved in deletion formation. While it is feasible that these pathways operate separately, crosstalk between replication and repair could also contribute to the establishment of deleted mtDNA molecules (Figure 5). This crosstalk has been demonstrated previously as a replication-dependent repair model as the basis for CD formation (38). In this model, deletions form by replication fork stalling near the deletion break site, followed by mispairing of the 13 bp repeats to form a loop structure that evokes the mitochondrial repair machinery (Figure 2B). The recruitment of repair proteins, following replication stalling, is thought to degrade the looped structure; thus suggesting a replication-repair crosstalk mechanism (Figure 5). After degradation of the loop, a ligation step to join the ends would recircularize mtDNA to form the deleted molecule. Several open questions remain to elucidate the source of the replication barrier leading to stalling. For example, is the origin of this barrier more related to nucleotide modifications or motifs in the mtDNA or rather in dysfunctional replisomal proteins that are damaged or mutated? Further, it would be interesting to test the role of repeats in deletion formation; the recent study demonstrating base editing in mtDNA (139) provides a new approach to manipulate the mitochondrial genome and answer these fundamental questions. Interestingly, copy-choice recombination could also explain the formation of the CD proposed by the replication-repair crosstalk model, where only breaks on the H-strand near the 3′-end of the 13 bp repeat by OriL triggered deletion formation. However, copy-choice recombination is challenging to envision for deletions that remove OriL (140). Thereby, suggesting an alternative mechanism of deletion formation in the case of this specific, albeit rare, type of deletion.

## CONCLUSION

Deletions in mtDNA ablate key genes that contributes to perturbed mitochondrial bioenergetics observed in several human diseases and mitochondrial disorders. While there is not a unified view on mtDNA deletion formation, aberrant replication and repair pathways are central to this process. In this review, we propose that while replication and repair could contribute to deletions via distinct pathways, they also could operate in a crosstalk mechanism whereby molecular players from both pathways are employed. To further understand the molecular basis of deletion formation, further work is needed to elucidate how mtDNA damage, secondary structures like G4s, and repeat sequences promote these mutations. Overall, there are limited mechanistic studies characterizing mtDNA deletion formation and it remains a poorly understood process. Until we have narrowed this gap, it is unlikely that we will be able to effectively treat diseases associated with mtDNA deletions.
